# Monoacylglycerol Analysis Using MS/MS^ALL^ Quadruple Time of Flight Mass Spectrometry

**DOI:** 10.3390/metabo6030025

**Published:** 2016-08-17

**Authors:** Fei Gao, Justice McDaniel, Emily Y. Chen, Hannah Rockwell, Matthew D. Lynes, Yu-Hua Tseng, Rangaprasad Sarangarajan, Niven R. Narain, Michael A. Kiebish

**Affiliations:** 1 BERG, LLC, 500 Old Connecticut Path, Bldg B, 3rd Floor, Framingham, MA 01701, USA; Fei.Gao@Berghealth.com (F.G.); Justice.McDaniel@Berghealth.com (J.M.); Emily.Chen@Berghealth.com (E.Y.C.); Hannah.Rockwell@Berghealth.com (H.R.); Rangaprasad.Sarangarajan@Berghealth.com (R.S.); Niven.Narain@Berghealth.com (N.R.N.); 2 Section on Integrative Physiology and Metabolism, Joslin Diabetes Center, Harvard Medical School, Boston, MA 02215, USA; Matthew.lynes@joslin.harvard.edu (M.D.L.); yu-hua.tseng@joslin.harvard.edu (Y.-H.T.)

**Keywords:** monoacyglycerols, MS/MS^ALL^, mass spectrometry, QTOF, acylglycerols

## Abstract

Monoacylglycerols (MAGs) are structural and bioactive metabolites critical for biological function. Development of facile tools for measuring MAG are essential to understand its role in different diseases and various pathways. A data-independent acquisition method, MS/MS^ALL^, using electrospray ionization (ESI) coupled quadrupole time of flight mass spectrometry (MS), was utilized for the structural identification and quantitative analysis of individual MAG molecular species. Compared with other acylglycerols, diacylglycerols (DAG) and triacylglycerols (TAG), MAG characteristically presented as a dominant protonated ion, [M + H]^+^, and under low collision energy as fatty acid-like fragments due to the neutral loss of the glycerol head group. At low concentrations (<10 pmol/µL), where lipid-lipid interactions are rare, there was a strong linear correlation between ion abundance and MAG concentration. Moreover, using the MS/MS^ALL^ method the major MAG species from human plasma and mouse brown and white adipose tissues were quantified in less than 6 min. Collectively, these results demonstrate that MS/MS^ALL^ analysis of MAG is an enabling strategy for the direct identification and quantitative analysis of low level MAG species from biological samples with high throughput and sensitivity.

## 1. Introduction

**Figure 1:**
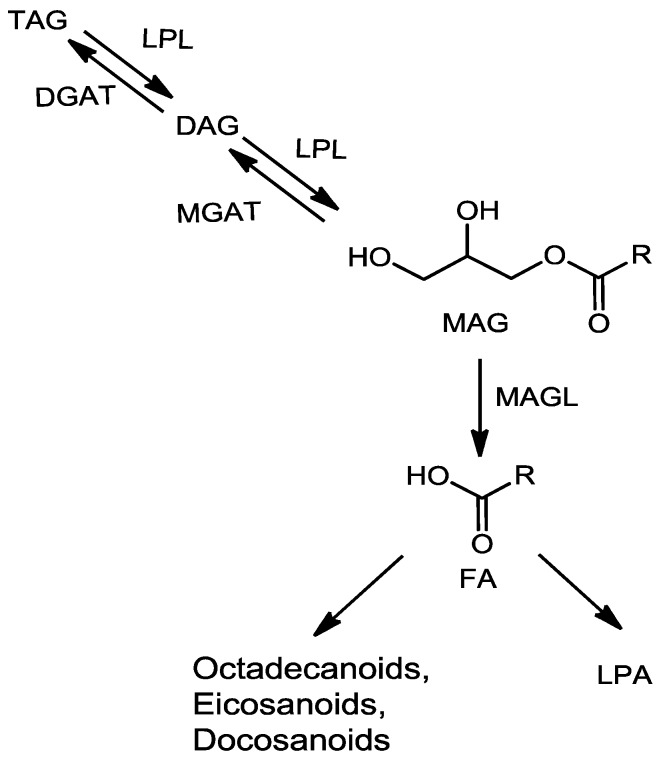
Biological pathways of monoacylglycerol (MAG). LPL, lipoprotein lipase; DGAT, diacylglycerol acyltransferase; MGAT, monoacylglycerol acyltransferase; MAGL, monoacylglycerol lipase; DAG, diacylglycerol; TAG, triacylglycerol; LPA, lysophosphatidyl acid; FA, fatty acid.

Monoacylglycerol (MAG) is a glyceride in which a glycerol molecule forms an ester bond with exactly one fatty acid (FA) molecule in either sn-1 or sn-2 positions. MAG is either biosynthesized as an intermediate during lipogenesis or formed by the hydrolysis of fat (mainly of triacylglycerols) (Figure 1). MAG plays an important role in lipid metabolism and homeostasis, as it is positioned in the center of the lipid pathways and controls the flow and distribution of fatty acids to other complex lipids, such as triacylyglycerols (TAG) and phospholipids [1]. The level of MAG in any biological system is tightly regulated by several enzymes, in particular monoacylglycerol lipase (MAGL). Notably, the disruption of MAGL can perturb the balance of monoglycerides, including the endocannabinoid 2-arachidonoyl [2,3], and even promote cancer pathogenesis [4].

MAGs are mostly found at very low levels in human plasma and organ tissues. As such, quantitative analysis is a considerable challenge as it requires highly sensitive methods for detection. Traditionally, the analysis of MAG is carried out using gas chromatography (GC) and gas chromatography mass spectrometry (GC-MS) [5,6,7] after derivatization, or reversed-phase HPLC with a UV detector [8]. However, separation and derivatization is time-consuming, and thus does not meet high throughput requirements for application using clinical samples. Moreover, the complexity of matrix and lipid species further requires tailored analytical techniques to accurately quantify MAG species in various sample types.

Due to recent advances in mass spectrometry and ionization technology, the molecular species of lipids can be analyzed by electrospray ionization-mass spectrometry (ESI-MS) coupled with HPLC separation [9,10]. MAG usually forms [M + H]^+^ or adduct ions, which can be further fragmented into fatty acid related ions for structural elucidation. Under a tandem mass spectrometry strategy, lipid classes, such as glycerophospholipids, glycerolipids, and sphingolipids, can be identified based on their characteristic fragmented ion. Traditionally, these types of ions have been used in precursor ion scanning, neutral loss scanning, and multiple reaction monitoring experiments, to measure the lipid molecular species, coupled with either direct infusion, e.g., shotgun lipidomics, or liquid chromatography (LC) modes, mainly on triple quadrupole (QQQ) instruments [11,12]. However, the drawbacks of such analysis are that they are highly targeted and they require the pre-selection of specific ions. Currently, there are two alternative strategies for global lipid determination: data-dependent acquisition (DDA) and data-independent acquisition (DIA). DDA is typically based on surveying an MS scan and involves user-defined information dependent criteria to select the precursor ions for MS/MS fragmentation. DDA allows the identification of unknown lipid molecular species and is usually coupled with an LC system. However, the analysis of global lipidomics using DDA has some limitations, such as poor reproducibility, limited dynamic range, and bias towards high abundance lipid species [13,14,15]. DIA overcomes these limitations by parallelizing the fragmentation of all detectable ions within a certain *m*/*z* range, providing a broader dynamic range, higher reproducibility, and better sensitivity [13].

Herein, we report a high throughput and highly sensitive MS/MS^ALL^ workflow for profiling MAG molecular species via the direct infusion of lipid extracts on a quadrupole time of flight mass spectrometer [13,16]. MS/MS^ALL^ is a direct infusion DIA technique, specifically designed for global lipidomics [13,16]. In this report, all precursor ions in the Q1 quadrupole at 1 Da *m*/*z* window were selected. MS/MS fragmentation was then carried out in Q2, and TOF collected all the high resolution MS/MS spectra at a high speed. These conditions and parameters can also be performed on other Q-TOF or hybrid orbitrap mass spectrometers with the correct software and hardware parameters, which thus allows for broad utilization of the method described here. For MAG species, the ionization and fragmentation conditions were optimized using 17:1 MAG as a standard and compared to two common acylglycerols, diacylglycerol (DAG) and triacylglycerol (TAG). The approach presented here demonstrates robust quantification of low level MAGs in human plasma and mouse brown and white adipose tissues in less than 6 min with a wide dynamic range, high throughput, and high sensitivity.

## 2. Results

### 2.1. Characterization MAG, DAG, and TAG Ionization Efficiency

**Figure 2:**
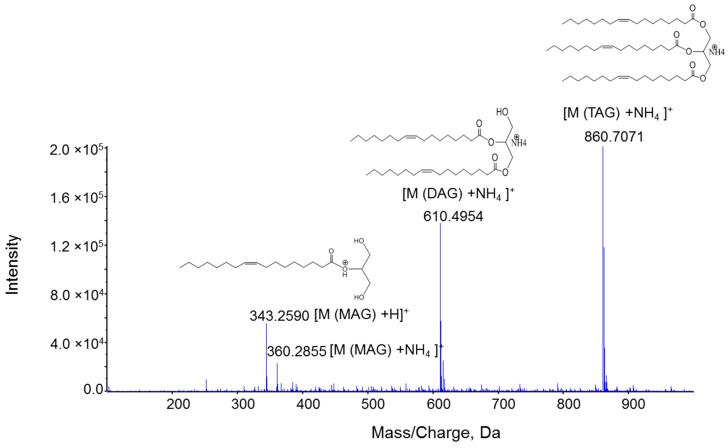
ESI-MS spectra for equal molar concentration (1 µM) of MAG 17:1, DAG d17:1, and TAG t17:1. Spectra were acquired with a TripleTOF 5600+ under the following conditions: GS1 = 10, GS2 = 10, CUR = 15, Temp = 300 °C.

To optimize the ESI-MS and MS/MS ionization efficiency of MAG compared to other acylglycerols DAG and TAG using MS/MS^ALL^ analysis, a mixture of equal molar concentration (1 µM) of MAG 17:1, DAG d17:1, and TAG t17:1 was infused into the mass spectrometer to optimize and compare the ionization and fragmentation patterns of these three acylglycerides. Non-polar lipids such as acylglycerides easily form adduct ions, so 2 mM ammonium acetate was added into the mobile phase (isopropanol:methanol:acetonitrile:H_2_O (3:3:3:1, by vol.)), to facilitate the formation of ammonium adduct ions [M + NH_4_]^+^. Under the experimental conditions, as expected, ammonium adducts of DAG d17:1 and TAG t17:1 appeared as dominant molecular ions, whereas the ionization of MAG 17:1 showed two forms of molecular ions with the major protonated ion at *m*/*z* 343.2 and the minor ammonium adduct ion at *m*/*z* 360.3 (Figure 2).

**Figure 3:**
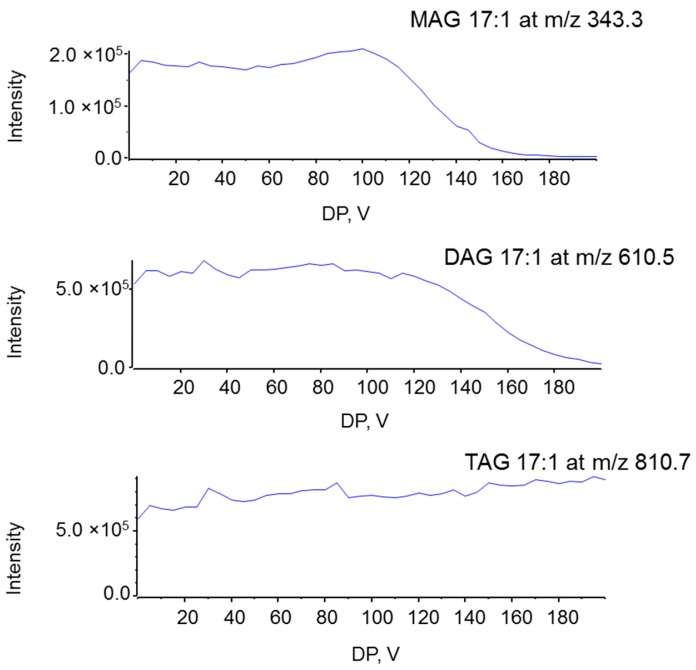
Effects of the declustering potential (DP) on ESI ionization of MAG 17:1, DAG d17:1, and TAG t17:1 at a concentration of 1 µM. Spectra were acquired under the following conditions: GS1 = 10, GS2 = 10, CUR = 15, Temp = 300 °C.

In order to yield stable and highly sensitive MS and tandem MS signals for MAG and other acylglycerols the QTOF parameters were optimized. The declustering potential (DP) is a voltage applied to the orifice that helps prevent ions from clustering, but high DP can introduce the in-source fragmentation [17]. Thus, the DP value affects the ionization efficiency and sensitivity, which thus needs to be optimized. In these experiments, the DP was ramped from 0 to 200 volts (V) with a 2 V step. The intensities of the molecular ions of MAG 17:1, DAG d17:1, and TAG t17:1 were recorded. Figure 3 shows the relatively stable ionization curve of all three acylglycerides with a DP at 60–100 V. At DP above 120 V, the intensity of MAG 17:1 and DAG d17:1 decreased gradually while the intensity of TAG t17:1 continued to increase. Considering other impurities in the solutions, we thus selected a DP of 100 V for our next experiments.

**Figure 4:**
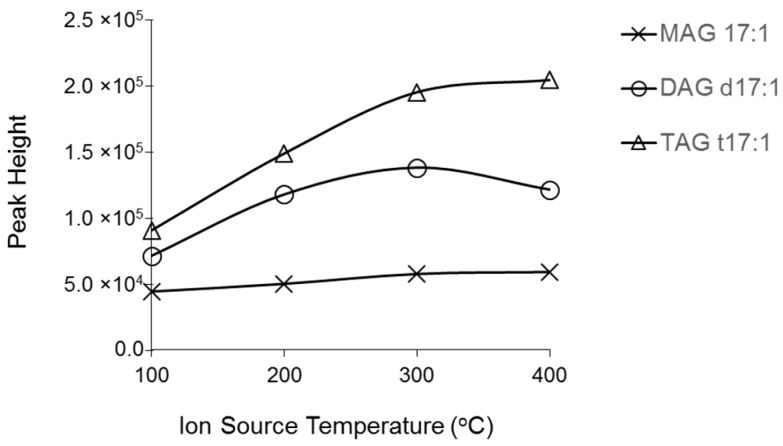
Effects of ESI source temperature on ESI ionization of MAG 17:1, DAG d17:1, and TAG t17:1 at a concentration of 1 µM. Spectra were acquired under the following conditions: GS1 = 10, GS2 = 10, CUR = 15, DP = 100 V.

Another parameter that affects ionization efficiency and sensitivity of compounds in ESI-MS experiments is ion source temperature. Higher temperatures help accelerate the evaporation process during ESI, but can result in hydrolysis of labile compounds. We performed experiments with a constant infusion of a mixture of MAG 17:1, DAG d17:1, and TAG t17:1 at temperatures that increased from 100 to 400 °C. When the temperature was increased from 100 to 300 °C, ionization efficiency, expressed as peak height of molecular ions, increased linearly (Figure 4). However, peak height leveled off at 400 °C for DAG and TAG. Given the stability of compounds at lower temperatures, 300 °C was selected as the ion source temperature for further optimization.

### 2.2. Fragmentation Mechanism of MAG, DAG, and TAG

**Figure 5:**
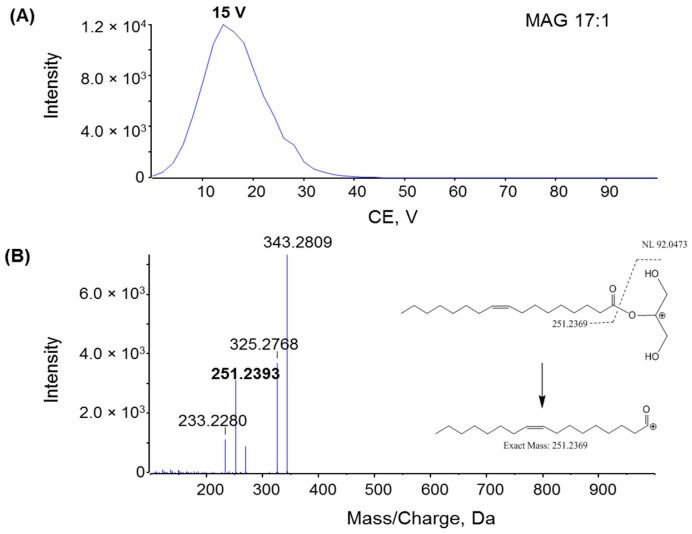
(**A**) Plot of intensity of fragment *m*/*z* 251.2393 of MAG 17:1 with varying rolling collision energy (CE); (**B**) MS/MS spectra for MAG 17:1 with CE = 15 V; insert: the fragmentation pathway of MAG 17:1 via ESI tandem MS. Spectra were acquired under the following conditions: GS1 = 10, GS2 = 10, CUR = 15, Temp = 300 °C, DP = 100 V, CE = 15 V.

**Figure 6:**
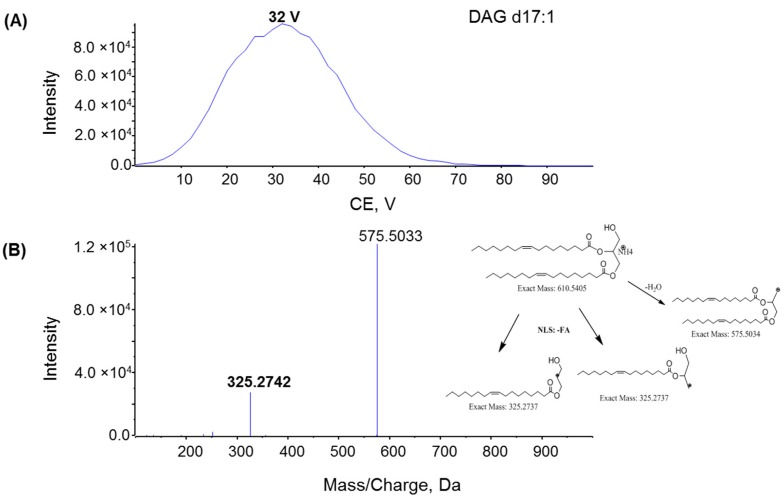
(**A**) Plot intensity of fragment *m*/*z* 325.2742 of DAG d17:1 with varying rolling collision energy (CE); (**B**) MS/MS spectra for DAG d17:1 with CE = 32 V; insert: the fragmentation pathway of DAG d17:1 via ESI tandem MS. Spectra were acquired under the following conditions: GS1 = 10, GS2 = 10, CUR = 15, Temp = 300 °C, DP = 100 V, CE = 32 V.

**Figure 7:**
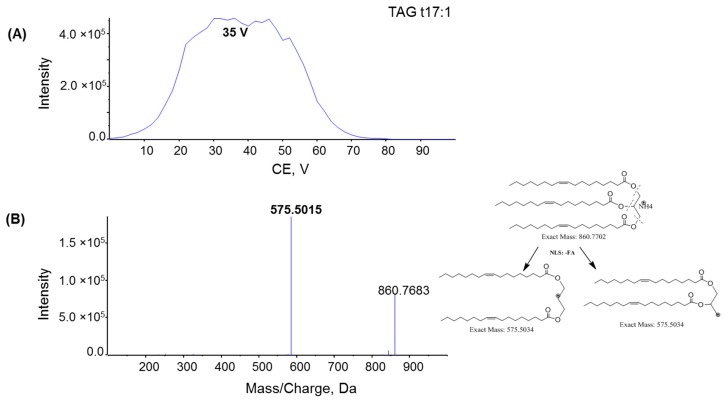
(**A**) Plot intensity of fragment *m*/*z* 575.5015 of TAG t17:1 with varying rolling collision energy (CE); (**B**) MS/MS spectra for TAG t17:1 with CE = 38 V; insert: the fragmentation pathway of TAG t17:1 via ESI tandem MS. Spectra were acquired under the following conditions: GS1 = 10, GS2 = 10, CUR = 15, Temp = 300 °C, DP = 100 V, CE = 35 V.

To investigate the fragmentation mechanism and improve product ion sensitivity, MS/MS fragmentation of MAG 17:1, DAG d17:1, and TAG t17:1 were performed under different collision energies (CE) ranging from 0 to 100 V. The dominant product ions from each compound were monitored during infusion. The optimal CE for the product ion of MAG 17:1 was 15 V (Figure 5A), whereas the optimal CE for the product ions of DAG and TAG was 32 V and 35 V, respectively (Figure 6A and Figure 7A). The product ion MS/MS spectra for MAG 17:1, DAG d17:1, and TAG t17:1 were then acquired at each optimal CE and the possible fragmentation pathways were analyzed. The MS/MS spectrum of MAG 17:1 resulted in the dominant product ion at *m*/*z* 251.2393 as [FA − H_2_O + H]^+^ due to the neutral loss of glycerol (Figure 5B), whereas for corresponding DAG and TAG, the dominant product ions [M − FA]^+^ resulted from the neutral loss of one fatty acid chain (Figure 6B and Figure 7B, respectively).

### 2.3. Quantification of MAG Using the MS/MS^ALL^

**Figure 8:**
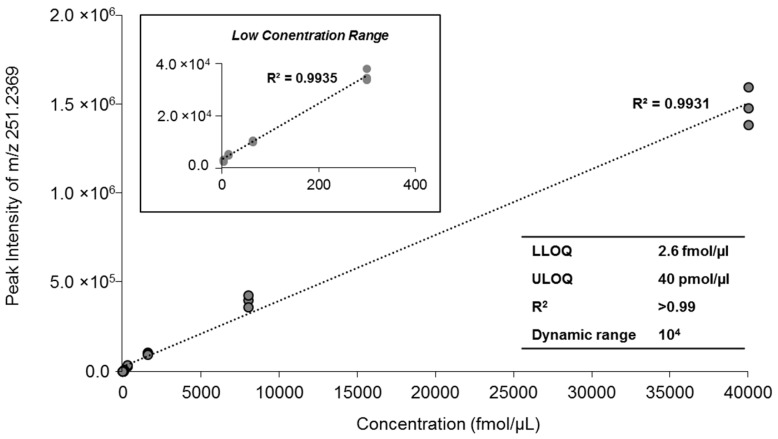
The plot of ESI-MS/MS response of MAG vs. the concentration of infused MAG 17:1 yielded a linear line (R^2^ = 0.9993) when spiked in human plasma. The insert table shows the related quantitative parameters. LLOQ, the lower limit of quantification. The full high-resolution MS and MS/MS data were collected using the MS/MS^ALL^ mode under the following conditions: GS1 = 10, GS2 = 10, CUR = 15, Temp = 300 °C, DP = 100 V, CE = 15 V ± 10 V.

**Figure 9:**
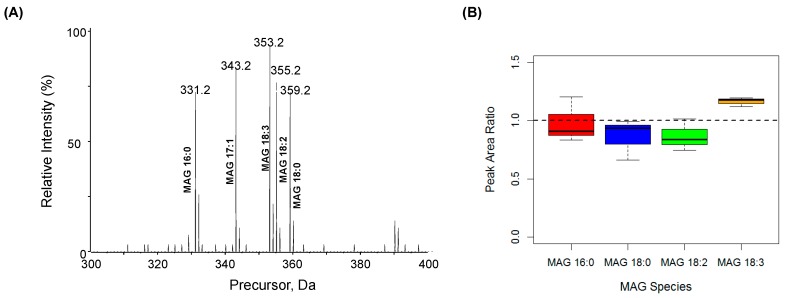
(**A**) Neutral loss (*m*/*z* 92.0473) tandem mass spectra of an equimolar mixture (10 fmol/µL); (**B**) The peak area ratio of individual MAG species to MAG 17:1 (*n* = 3). The full high-resolution MS and MS/MS data were collected using the MS/MS^ALL^ mode under the following conditions: GS1 = 10, GS2 = 10, CUR = 15, Temp = 300 °C, DP = 100 V, CE = 15 ± 10 V. The neutral loss scan MS/MS spectra was generated from the collected data retrospectively.

High-resolution analysis of fragment ions using the MS/MS^ALL^ strategy was used for accurate quantification as previously reported [16,18]. To evaluate the linear response of MAG and its dynamic quantification range based on the MAG-derived fragment ions, we prepared a serial dilution of MAG 17:1 in human plasma and plotted the calibration curves ranging from 2.6 fmol/µL to 40 pmol/µL, with an R^2^ regression value of 0.9931 (Figure 8). To assess the reproducibility, we monitored the response of the synthetic standard, MAG 17:1, with different concentrations in a series of three replicates of human plasma. The intensity of characteristic fragment m/z 251.2393 resulted in a coefficient of variation (CV) in the range of 2%–8.5%, except at the lower limit of quantification (LLOQ) concentration where the CV was 13.2% (Table 1). The low concentration of lipids in the infusion solution is essential for accurate quantification of lipid molecular species with the same polar head group because at low concentrations the ionization efficiency of lipids is determined by the polar head group and is independent of fatty acyl chain length and unsaturation [11,12]. To confirm this for MAG, equal molar MAGs with different fatty acid chain lengths and numbers of double bonds were profiled based on the neutral loss tandem MS spectrum at *m*/*z* 92.0473 (spectrum was obtained from the acquired MS/MS^ALL^ data). All MAG species with different chain length and double bonds showed nearly identical ionization efficiency (Figure 9A) and the ratio of the MAG species to MAG 17:1 was around 1 (Figure 9B). For biological samples, selected concentrations of the internal standard, MAG 17:1, were spiked into plasma or adipose tissue homogenates before extraction. Sample preparation was performed as described in the materials and methods, and the samples were diluted in the running buffer to ensure the dynamic range and ionization efficiency. Another prerequisite for accurate quantification for MS/MS^ALL^ or any other data independent acquisition (DIA) is the database searching and accurate quantitation based on optimal ion/adduct selection. An in-house database for MAGs was constructed based on the information of MAG high resolution m/z of molecular ions and diagnostic fragments (Table 2).

**Figure 10:**
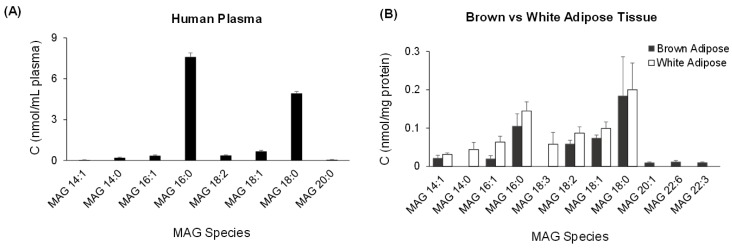
Profile of MAG molecular species obtained from (**A**) human plasma and (**B**) mouse brown and white adipose tissue samples. The data represent means ± S.E. of *n* = 6 mice and 6 replicates of human plasma. The full high-resolution MS and MS/MS data were collected using the MS/MS^ALL^ mode under the following conditions: GS1 = 10, GS2 = 10, CUR = 15, Temp = 300 °C, DP = 100 V, CE = 15 ± 10 V. The neutral loss scan MS/MS spectra was generated from the collected data retrospectively.

The DIA MS/MS^ALL^ method was applied to analyze the profile of MAGs in three representative sample types that would either be routinely used or commonly investigated for lipid metabolism. Human plasma and mouse brown and white adipose tissues were selected for analysis using the MS/MS^ALL^ method. The dominant MAG species in human plasma were saturated MAG 18:0 and MAG 16:0, others consisted of medium-chain fatty acids. Compared to mouse white adipose tissue, brown adipose tissue showed a distinct pattern of MAG with notably more polyunsaturated fatty acid MAGs, such as MAG 22:3 and MAG 22:6 (Figure 10).

## 3. Discussion

MAGs are found in trace amounts in blood and tissues and play an essential role in metabolic homeostasis and diverse physiological functions [3,4,7]. Mass spectrometry based platforms have advanced significantly in the past few years, providing opportunities to develop robust high throughput analysis of MAGs in diverse sample types with improved sensitivity and identification. Herein, we have adapted and optimized a DIA MS/MS^ALL^ shotgun lipidomics method for the analysis of MAGs that can be utilized for various biological samples and for broader use in defining the role of metabolic intermediates in regulating physiological function.

Although MAG, DAG, and TAG all belong to the acylglycerol family, which are the most abundant neutral lipids in mammals, their ESI-MS behavioral properties are quite different. MAG with only one fatty acid chain preferentially ionizes to form [M + H]^+^ as the predominant molecular ion even in the presence of ammonium acetate, whereas DAG and TAG with two and three fatty acid chains, respectively, easily form ammonium ion adducts. Also, the ratio of the intensity of molecular ions is roughly proportional to the number of fatty acid chains (1:2:3). The different ESI-MS behavior of acylglycerols demonstrates that the molecular structure of the acylglycerols can influence its ESI behavior. Furthermore, the hydrophobic portion of acylglycerol can determine the ESI response because analytes with greater hydrophobicity have higher affinities for the surface of ESI droplets, which in turn produces higher ESI signals [19]. In addition, the different molecular structure of acylglycerols and the composition of the ESI solution influences the MS/MS fragmentation pathways and mechanism [9,11,12,20]. Alkali metal adduct ions of acylgylcerols usually produce the specific fragments due to the neutral loss of fatty acids [9,12]. Our results are consistent with DAG and TAG ammonium adduct molecular ions, whereas MAG displayed a different fragmentation pattern due to the neutral loss of glycerol.

Shotgun lipidomics exploits the chemical and physical properties of lipids to facilitate the high throughput global MS-based analysis of a lipidome directly from organic extracts of biological samples [12,21]. The lipidome is profiled based on the specific head groups and fatty acid chains either through precursor ion scans, neutral loss scans, or multiple reaction monitoring experiments, primarily carried out on triple quadrupole (QQQ) instruments [12,22,23]. However, the disadvantage of QQQ analyses is that they require the pre-selection of characteristic ions and are highly targeted due to the vast number of theoretical ions that have to be pre-selected. Instead, data independent acquisition methods, such as the MS/MS^ALL^ technique, have proven successful for untargeted lipid analysis [13,16,18]. A key point in MS/MS^ALL^ analysis is selection of the optimal collision energy of molecular ions to produce the most abundant distinct fragment ions in each window. For accurate quantification of MAGs and other lipids using the MS/MS^ALL^ strategy, similar to all other the shotgun lipidomics methods [11,24,25], it is favorable to quantitate lipids when at a low concentrations in solution as at low concentrations lipid-lipid interactions are negligible and ionization efficiency of the molecular species of the same lipid class is predominantly determined by the nature of the polar head group. For MAG, we illustrated at a low concentration of 10 fmol/µL different MAG species of various chain lengths and number of double bonds had similar peak intensities and areas under the optimal ESI/MS conditions. The linear range for quantification of MAGs was between the fmol/µL and pmol/µL range. The low concentration range requires the appropriate dilution of the lipid extracts for accurate quantification [11,12,16]. One limitation of the MS/MS^ALL^ method for MAG analysis is that it does not differentiate the regio-isomers, i.e. the sn-position of the fatty acid chain in the glycerol backbone. This can be resolved by using gas chromatography, liquid chromatography, or ion mobility coupled to mass spectrometry [7,10,26].

The MS/MS^ALL^ method for MAG was successfully applied as demonstrated by the results from MAG profiling of human plasma and mouse white and brown adipose tissues. Saturated fatty acid MAG species, such as MAG 18:0 and MAG 16:0 dominate MAG components in all three samples. It is notable that white and brown adipose tissue showed differences in their pattern of MAG species, which may explain functional differences between them. For example, white and brown adipose tissues differ significantly based on their structure and physiology [27,28]. Specifically, the primary purpose of white adipose tissue is to store and release energy in the form of free fatty acids, while the essential function executed by brown adipose tissue is the expenditure of fatty acid-derived energy for maintenance of the organism’s thermal stability [29,30,31]. Our MAG analysis showed a similar distribution for a large amount of fatty acids, such as MAG 16:0, MAG 18:0, MAG 18:1, and MAG 18:2, between white and brown adipose tissue. These MAG species can be immediately accessed by enzymes and provide the lipid source for either energy needs or thermal balance [32]. Interestingly, in brown adipose tissue, there were certain long-chain polyunsaturated fatty acids, such as MAG 22:6 and MAG 22:3, which may be released by monoacyglycerol lipase and converted into bioactive lipid mediators.

In summary, we established an MS/MS^ALL^ method for accurate identification and quantification of MAG species from complex biological samples through the optimization of ESI/MS parameters. Compared with other acylglycerols, DAG and TAG, MAG demonstrated distinct MS and tandem MS ionization conditions, which were exploited for MAG identification and quantification in the biological samples. The method developed here can be applied to broader studies for measurement of MAG in biofluids and diverse sample types in a robust and high throughput approach.

## 4. Experimental Procedures

### 4.1. Materials

MAG 17:1, MAG 16:0, MAG 18:0, MAG 18:2, MAG 18:3, DAG d17:1, and TAG t17:1 were purchased from Nu-Chek Prep Inc (Waterville, MN, USA). All solvents were of HPLC or LC/MS grade and were purchased from Fisher Scientific (Waltham MA, USA) or VWR International (Radnor, PA, USA). Human plasma was purchased from Bioreclammation (Baltimore, MD, USA) and mouse brown and white adipose tissues were obtained from Joslin Diabetes Center. All animal procedures were approved by the Institutional Animal Use and Care Committee at Joslin Diabetes Center.

The stock solutions of all acylglycerols were prepared using chloroform:methanol (1:1, *v*/*v*) and the working solutions were prepared by diluting the stock solutions using the running buffer (see below).

### 4.2. Sample Preparation and Extraction

Human plasma samples were thawed on ice. Before extraction, an appropriate amount (1.65 nmol) of the internal standard, MAG 17:1 was added into 25 µL of plasma. Then, 4 mL chloroform:methanol (1:1, *v*/*v*) and 2 mL LiCl solution (50 mmol) were added to each sample and the lipid extraction was performed as previously described [16,33]. Specifically, the extraction homogenate was vortexed and centrifuged at 2500 x g for 5 min. The chloroform layer of each extract mixture was carefully removed and collected. An additional 2 mL chloroform was added into the MeOH/aqueous layer of each test tube. After centrifugation, the chloroform layer from each individual sample was combined and dried under a nitrogen stream. Each individual residue was then re-suspended in 4 mL chloroform/methanol (1:1), back-extracted against 2 mL LiCl aqueous solution (10 mmol), and the extract was dried as described above. Such lipid extraction was automated using a customized sequence on a Hamilton Robobtics STARlet system (Reno, NV, USA) to meet the high throughput requirements. Finally, lipid extracts were dried under nitrogen and reconstituted in 68 µL chloroform:methanol (1:1, *v*/*v*). Samples were flushed with nitrogen and stored at −20 °C.

To investigate the dynamic quantification range and assay reproducibility, the synthetic standard, MAG 17:1, was spiked at different serial concentrations into 25 µL human plasma prior to extraction and experiments were performed in triplicates.

For tissue analysis, 10–20 mg of mouse adipose tissue was transferred to an OMNI bead tube with 0.8 mL 10× diluted PBS and was homogenized for 1.5 min at 4 °C using the OMNI Cryo Bead Ruptor 24 (OMNI international, Inc., Kennesaw, GA, USA). An aliquot of the homogenate was taken for protein analysis using a BCA assay and 1 mg of sample based on protein concentration was added to internal standards and 4 mL chloroform:methanol (1:1, *v*/*v*) for robotic extraction [34]. Lipid extracts were dried under nitrogen and reconstituted in 400 µL chloroform:methanol (1:1, *v*/*v*). Samples were flushed with nitrogen and stored at −20 °C.

### 4.3. Direct Infusion Quadruple Time of Flight (QTOF) Mass Spectrometry

The concentrated samples (both plasma and tissue) were diluted 50× in isopropanol: methanol:acetonitrile:H_2_O (3:3:3:1, by vol.) with 2 mM ammonium acetate in LC vials, and 50 µL diluted lipid extract was automatically loaded and directly delivered to the ESI source using an Ekspert microLC 200 system (SCIEX, Framingham, MA, USA) with a flow rate of 6 µL/min on a customized sample loop. The MS/MS^ALL^ acquisition experiment was performed on a SCIEX TripleTOF 5600+ (SCIEX, Framingham, MA, USA). ESI source parameters included nebulizing gases (GS) GS1 at 10, GS2 at 10, curtain gas (CUR) at 15, positive mode ion spray voltage at 5000, declustering potential at 100 V, and temperature 300 °C. The atmospheric-pressure chemical ionization (APCI) probe and inlet were connected to a calibrant pump which delivers mass calibration solution for MS and MS/MS. The MS/MS^ALL^ data acquisition was controlled with Analyst^®^ TF 1.5.1 software (SCIEX). An MS range was 200–1200 *m*/*z* at an accumulation time of 300 ms, followed by 1000 product ion scans with 1000 precursors evenly spaced from *m*/*z* 200.051 to *m*/*z* 1200.051, measuring across *m*/*z* 100–1500 with the accumulation time 300 ms each. Total time for one MS/MS^ALL^ acquisition was 5.5 min. The optimal collision energy (CE) for MAG MS/MS fragmentation was 15 ± 10 eV.

## Figures and Tables

**Table 1:** Reproducibility of the MS/MS^ALL^ technique for MAG.

Concentration	Peak Intensity			Mean (*n* = 3)	CV (%)
	Sample A	Sample B	Sample C		
40 pmol/µL	1,360,500	1,454,000	1,568,500	1,461,000	7.13
8 pmol/µL	397,250	421,500	356,450	391,733	8.39
1.6 pmol/µL	105,950	105,590	94,170	101,903	6.57
0.3 pmol/µL	33,190	32,400	36,615	34,068	6.58
64 fmol/µL	9840	9464.5	9841.5	9715	2.24
13 fmol/µL	4562.5	4839	5110	4837	5.66
2.6 fmol/µL	2323	2807	3020	2716	13.2

**Table 2:** MAG Species Database.

MAG Species	[M + H]^+^	Diagnostic Fragment [FA + H − H_2_O]^+^
MAG 14:1	301.3	209.1906
MAG 14:0	303.3	211.2062
MAG 16:1	329.3	237.2219
MAG 16:0	331.3	239.2376
IS MAG 17:1	343.3	251.2369
MAG 18:3	353.3	261.2219
MAG 18:2	355.3	263.2376
MAG 18:1	357.3	265.2532
MAG 18:0	359.3	267.2689
MAG 20:4	379.3	287.2376
MAG 20:3	381.3	289.2532
MAG 20:2	383.3	291.2689
MAG 20:1	385.3	293.2845
MAG 20:0	387.4	295.3002
MAG 22:6	403.3	311.2376
MAG 22:5	405.3	313.2532
MAG 22:4	407.3	315.2689
MAG 22:3	409.3	317.2845
MAG 22:2	411.4	319.3002
MAG 22:1	413.4	321.3158
MAG 22:0	415.4	323.3315
